# Improvements in physical activity and depression symptoms: an observational study of users of a multi-modal digital mental health platform

**DOI:** 10.3389/fdgth.2025.1394647

**Published:** 2025-11-06

**Authors:** Camille E. Welcome Chamberlain, Shannon Lindsay, Brooke J. Smith, Sara Sagui Henson, Cynthia Castro Sweet, Sara M. Levens

**Affiliations:** 1 Clinical Strategy and Research Team, Modern Health, San Francisco, CA, United States; 2 Department of Psychological Science, University of North Carolina at Charlotte, Charlotte, NC, United States

**Keywords:** digital health, mental health symptoms, physical activity, depression, technology

## Abstract

**Background:**

Moderate-to-vigorous physical activity (MVPA) and depression symptoms have a longstanding, inverse relationship. This short-term study examined the patterns of MVPA and prevalence of depression symptoms among adults seeking care from an employer-sponsored, multi-modal digital mental health platform.

**Methods:**

Adults (*n* = 755) with access to the platform enrolled in an online, observational study over 3 months. Baseline and longitudinal analyses were conducted on self-report MVPA and depression symptoms. Rates of activity and symptom improvement and maintenance were evaluated.

**Results:**

Approximately 47% of participants were physically underactive at baseline, defined as <150 min of physical activity per week. Men participated in more physical activity than women (*p* = 0.005), while women and individuals identifying as gender non-binary reported more depression symptoms than men (*p*s < 0.05). Older adults reported fewer depression symptoms than younger adults (*r* = −0.16, *p* < 0.001). Baseline MVPA baseline was negatively correlated with depression symptoms (*r* = −0.19, *p* < 0.001). Depression scores significantly improved, with 66.9% of adults at risk of depression improving or recovering (*p* < 0.001) and 94% of adults with low depression symptoms maintaining this status over time (*p* = 0.004). Physical activity participation improved by 117% among individuals deemed underactive at baseline (*p* < 0.001), while individuals who were active at baseline maintained high levels of physical activity (*p* = 0.06).

**Conclusions:**

Adults with access to a multi-modal digital mental health platform reported significant beneficial changes in depression symptoms and physical activity participation. Digital mental health tools may have the potential to elicit positive change in physical health domains, as well as mental health.

## Introduction

Engagement in physical activity is linked to numerous aspects of mental health, including enhanced psychological well-being (1) and reduced depression and anxiety symptoms (2–4). When individuals do not meet suggested physical activity guidelines [defined as 150 + weekly minutes of moderate-to-vigorous aerobic physical activity (MVPA); (5)], they are more likely to experience poorer mental health outcomes, including depression symptoms (6). This relationship between physical activity and depression is well-documented. One investigation found that higher volumes of accelerometer-measured physical activity were associated with a reduced risk of depression, while another observed a detrimental effect of declining physical activity on depression scores over a 32-year period (7). Physical activity has antidepressant effects through biological (e.g., reduction in inflammation, increased resilience to oxidative stress) and psychological [e.g., improved mood and affect (8, 9), reduced stress and anxiety (9)] mechanisms (4), and is recommended as a step in treatment for individuals with mild-to-moderate depression or subclinical symptoms (10).

The relationship between physical activity and depression symptoms is bidirectional. Numerous longitudinal investigations have demonstrated that depression may be related to future sedentary behavior and/or physical inactivity (7, 11). In adolescents, prior depression symptoms (particularly affective symptoms) have been associated with an increased likelihood to report a reduction in physical activity at follow-up (12). This finding has also been echoed in investigations with adults (7, 13). Compared to individuals who are sufficiently active, insufficient physical activity is associated with an increased incidence of depressive episodes (14). Cumulatively this suggests that a decline in physical activity should be recognized as both a precursor to, and a consequence of, depression and its associated symptoms. Given these bidirectional effects, it is important to find novel ways to increase physical activity and/or decrease depression symptoms in a broader range of ways, such as using digital health and health technology.

Digital mental health is defined as the provision of psychological services and interventions through synchronous or asynchronous mediums online and/or on mobile platforms (15). In the field of digital mental health, platforms have traditionally focused on the provision of mental health services for the direct purpose of improving mental health outcomes and promoting mental wellness (16–19). Though clinically effective for mental health concerns (20, 21), these platforms have historically not targeted overlapping and interconnected behavioral and physical health domains and may overlook the indirect benefits of mental health care on adjacent behaviors such as physical activity. Higher levels of physical activity are associated with decreases in depression (14, 22), as well as increases in mental wellness and cognitive acuity (19, 23)—making it an ideal health behavior to promote on digital mental health platforms. Further, meta-analytic evidence supports physical activity as an effective supplemental tool for depression treatment (24, 25).

Digital mental health programs also provide the opportunity for tailored multi-modal support but have not been traditionally leveraged in this way. Digital health programs are often built to address one particular niche or health domain, such as weight loss, or as point solutions that focus on diabetes management, improving sleep hygiene, or practicing meditation and mindfulness (26). However, this siloed approach ignores the potential of digital solutions to simultaneously address multiple facets of health within the same platform. Health concerns are rarely isolated, as individuals often experience coexisting challenges across multiple domains of health and life (27, 28). Digital mental health programs should endeavor to provide comprehensive care that spans the entire spectrum of health and addresses several domains concurrently. Furthermore, digital mental health programs should also endeavor to provide a range of care modalities to effectively meet the varied needs and preferences of its users. Many digital mental health programs offer only one format of care, whether that is therapy, coaching, or digital resources. By doing so, these programs are limiting their reach and potential to improve multiple domains of health, as well as their ability to personalize care to diverse individuals (29).

The multi-modal digital mental health program in the present study is unique in its offerings, as it provides care across several domains through several modalities despite being primarily focused on mental health. Individuals provide their desired areas of focus (e.g., my physical well-being, my mental health) and care modality preferences (e.g., with a provider, with self-guided digital tools) and are recommended an initial care pathway based on this information. The platform offers individuals a range of care modalities they can access, including digital resources, online group psychoeducational sessions, one-on-one telecoaching sessions, and one-on-one teletherapy sessions, differing substantially from traditional models of mental health support and standard digital interventions (30, 31). As digital mental health platforms offer flexible support across a range of modalities, they are an ideal setting to examine the indirect benefits of mental health care on adjacent behaviors such as physical activity. Digital mental health platforms, and employee sponsored digital mental health platforms in particular, that offer support across a range of life domains (e.g., financial and mental health), may show improvements reflecting a behavior change synergy that can arise when individuals receive multi-modal support.

In this study, we examined the impact of a comprehensive employer-sponsored, multi-modal digital mental health platform offered as a healthcare benefit which provided multi-modal support across multiple health domains. The platform leverages technology to connect employees with a global network of licensed therapists and certified coaches and an extensive on-demand library of digital resources. All the evidence-based care modalities address health and well-being across five domains: emotional, social, physical, professional, and financial, meaning within the same platform a person could be working on their emotional health with a therapist, their physical health with a coach, and their professional well-being with self-guided digital resources. The platform uses employees' intake assessment data to connect them with the right care for their clinical needs and preferences. There is a paucity of data on the impact of a multi-modal digital mental health platform like this on different areas of health and well-being.

To contribute to literature on the link between mental and behavioral health within a single intervention, we examined levels of depression and physical activity in a real-world sample of employees with access to this digital mental health platform. Our investigation had three aims:

1. Analyze the prevalence of depression and levels of physical activity in employees seeking support from a digital mental health platform before starting care. This is crucial to advancing our understanding of real-world mental and behavioral health complexities and the importance of addressing multiple domains of health within a single platform.
2. Examine baseline associations between depression and physical activity to confirm prior research demonstrating their interaction.
3. Investigate changes in physical activity and depression over time to determine the impact of a digital mental health platform offering multi-modal care across several domains of health.

## Method

### Design and participants

This investigation was a prospective, observational study of individuals who recently sought services through an employer-sponsored, multi-modal digital mental health benefits platform [Modern Health, San Francisco, CA]. The study was conducted between September 2021 and May 2022. Eligible participants were 18 years or older, lived in the United States, had registered and onboarded for the mental health benefit, had access to a smartphone, tablet, or computer, had some initiation of mental health care through the platform (defined as either selecting a coach and/or therapist and/or engaging with at least one component of digital content), and provided informed consent to participate in an observational study. The study protocol was reviewed and approved by Western Clinical Group IRB.

### Procedures

Participants who created an account with the [Modern Health, San Francisco, CA] platform and passed an initial eligibility check using data from the platform (i.e., age, location, registration, platform initiation) were invited via email to complete a screening questionnaire. The questionnaire collected sociodemographic information (age, gender, race, ethnicity, highest level of education, and marital, family, caregiver, veteran, disability, and employment status) and organizational information (industry, size of company, employment setting, and job level). Recruitment outreach using the screening questionnaire responses was weighted across race, ethnicity, gender identities, age groups, and severity of mental health symptoms at baseline to ensure a sample representative of the platform's commercial population. Information about mental health diagnoses was not available. Participants provided informed consent and were e-mailed a secure link to the baseline survey and a follow-up survey three months later. All data were collected through a secure, online survey platform (Qualtrics, Provo, UT, USA). Participants were compensated with a $25 digital gift card for completion of each survey.

### Multi-modal digital mental health services

Eligible individuals registered for a platform account through a mobile app or website and completed initial clinical assessments to assess their level of clinical need (e.g., through standardized clinical measures like the PHQ-9 and GAD-7), desired health domain of focus (my emotions, my physical well-being, my relationships, my professional life, my finances) and care modality preferences (on my own, one-on-one, in a group, unsure). Based on the results of these assessments, a proprietary algorithm recommended an initial care pathway that considered their clinical acuity, what health domain they most wanted to work on, and how they wanted to receive care. Participants were able to self-refer and utilize any combination of care modalities, including one-on-one teletherapy sessions, one-on-one telecoaching sessions, self-guided digital resources, and online group psychoeducational sessions.

**Teletherapy and telecoaching.** Participants had access to 50-minute, one-on-one remote teletherapy services with licensed therapists with an advanced degree in clinical psychology or a related field (e.g., PhD, PsyD, LCSW, LMFT, or LPC) and skilled in evidence-based practices via a videoconferencing platform (Zoom). They also had access to 30 min, one-on-one remote telecoaching sessions, which were led by certified coaches and delivered via videoconferencing. All coaches had International Coaching Federation certification and at least 150 h of coaching experience. Coaches received additional training (at least 6 h) from the digital mental health company on topics like culturally centered care and how to identify high-risk situations necessitating escalation, as well as ongoing training through on-demand learnings and case consultations with licensed clinicians. All providers underwent a careful screening process by the digital mental health company and were vetted for offering evidence-based practices [drawn from Cognitive Behavioral Therapy (CBT), Acceptance and Commitment Therapy (ACT), Motivational Interviewing].

Ongoing provider quality monitoring and improvement was guided by a Quality Improvement Advisory Committee (a team of legal, compliance, provider operations, and clinical experts) and annual quality action plans driven by a Global Inclusion Council. Satisfaction and therapeutic alliance with providers on this platform have been high (e.g., 4.7+ out of 5) and reported in several previously published studies (32–34). The provider network coverage system leverages a global network of therapists and coaches and is designed to ensure that the platform can offer the quickest access to high-quality providers with an average time to the first available session being around one day. This system is supported by the platform's proprietary technology, which monitors real-time provider availability, capacity tracking, and proactive alerts for providers globally.

Participants could access teletherapy and telecoaching by being recommended to either of these care modalities after their intake assessment or by self-referral. In either case, based on their initial intake assessment, a proprietary algorithm matched them with a list of providers who specialized in the participants' health domain of focus, were fluent in their preferred language (55+ languages at the time of this study), and had session availability within a few days. The matching algorithm is designed to surface providers who are most relevant to a participant's clinical needs and preferences. It also considers provider factors like specializations, language, credentials, time zone, and availability which are critical to effective care delivery.

For example, if a participant wanted to work on their physical well-being, they were provided with a list of therapists or coaches who specialized in helping people meet their health goals (e.g., help a participant set and achieve fitness goals, create sustainable exercise routines, and integrate physical activity into their daily lives). In this health domain, telecoaches especially could work with participants to set realistic and personalized fitness goals, review progress, and address any obstacles that may hinder their exercise routines. The number of remote one-on-one provider sessions a participant attended depended on the sessions covered by their employer, their therapeutic need, and their level of engagement.

**Self-guided digital resources and group psychoeducational sessions.** All participants had unlimited access to an on-demand digital library of programs and resources across the five health domains that they could access at any time. These resources included mindfulness and meditation exercises, interactive programs, educational podcasts, self-paced structured educational lessons, and group psychoeducational sessions. All digital materials were developed and designed with the support of mental health experts including an in-house team of clinical psychologists. Digital health resources were designed to cover topics such as emotions, relationships, healthy lifestyles, professional life, and finances. The digital library contained several resources relevant to improving physical well-being, such as maintaining healthy lifestyles (e.g., mindful walking; mental benefits of physical exercise; take action: creating routines; full body stretch break; mid-day movement), navigating health challenges (e.g., physical wellness for resilience; working with discomfort), and sleep soundly (e.g., muscle relaxation; body scan for sleep; sleep hygiene; sleep and health).

Participants also had unlimited access to community-based spaces for learning and connection. Group psychoeducation sessions were live sessions led by licensed therapists and certified coaches from the platform network on a broad spectrum of topics ranging from foundational mental health subjects (e.g., emotional resilience) to global events (e.g., processing stressful world events) and community, identity-based, and affinity group sessions (e.g., POC men, Black individuals, Asian & Pacific Islander individuals). One series of group sessions focuses on physical well-being, for example a session called “Movement for Mental Wellness”. In this session, a provider focuses on the connection between physical activity and mental health and leads participants through a discussion of how physical activity can strengthen mental health, providing reminders to reflect and set intentions around physical activity. After initial creation, all sessions were audited by licensed clinicians to ensure that content was culturally sensitive and destigmatizing. Attendance was anonymous and session formats included webinar style learning, skill-building workshops, and informal discussions.

### Measures

#### Demographics

At baseline, participants provided age, gender identity, and race/ethnicity information through the screening questionnaire. Participants were able to select all that applied from a list of 11 gender identities and 7 racial and ethnic identities.

#### Physical activity

The International Physical Activity Questionnaire—Short Form (IPAQ) (35) measured volume of physical activity, including vigorous activity, moderate activity, walking, and sitting. Participants indicated the number of days per week and the average minutes per day they participated in these activities over the previous 7 days. We summed total time in moderate and vigorous activity to create a total MVPA (mins/week) variable to improve the interpretability of results. We also created a binary categorical variable to define participants as either “active” (engaging in ≥150 min/week of MVPA) or “underactive” (engaging in <150 min/week of MVPA), based on U.S. physical activity guidelines (5). The IPAQ has a moderate-to-high reliability and validity of 0.74 and 0.72, respectively (36, 37). Improvement was defined as having an MVPA score classified as underactive at baseline and active at follow-up. Maintenance was defined as having an MVPA score classified as active at both baseline and follow-up.

#### Depression symptoms

The Patient Health Questionnaire (PHQ-9) (38) assessed the presence and severity of depression symptoms over the past two weeks. Participants responded on a 4-point scale (0 = “Not at all”; 3 = “Nearly every day”). Total scores were summed to create a continuous score ranging from 0–27. Higher continuous scores indicate higher severity of depression symptomatology. We used validated clinical thresholds to create a binary variable to indicate if participants had a positive (PHQ-9 ≥ 10) or negative screening (PHQ-9 < 10) for depression. This established cut-off is often used in mental health outcomes research, as it maximizes both sensitivity and specificity (39, 40). We defined clinically significant improvement as a decrease in PHQ-9 scores by ≥5 points over the study period (39, 40). Recovery from depression was defined as having a positive screening for depressive symptoms at baseline and a negative screening at follow-up. Maintenance of good mental health was defined as having a negative screening for depressive symptoms at both baseline and follow-up.

#### Self-reported impact of mental health on physical activity

We constructed a single-item self-report measure to assess the perceived impact of one's mental health on their ability to participate in physical activity. This measure was based on item 20 of the 36-item Medical Outcomes Study (MOS) Short Form Survey (41). Participants were asked “During the past 4 weeks, to what extent have your mental health issues negatively impacted your engagement in physical activity?” on a 5-point scale (1 = “Not at all”; 5 = “Extremely”). Higher scores indicate greater perceived negative impact of mental health on physical activity participation.

#### Platform engagement

To assess utilization of the platform, we reported average engagement with each of the digital mental health modalities (e.g., number of teletherapy sessions). Data were not available for engagement in the group psychoeducational sessions as registration for those was anonymous. We defined overall engagement with the platform as attending at least one session with a teletherapist or 1 session with a telecoach or using at least one self-guided digital resource during the 3-month study period. If a participant did not engage in any care, we classified them as not engaged.

### Data analysis

Physical activity data were handled using the following criteria: (1) cases with missing IPAQ data were excluded from analyses, (2) cases with moderate or vigorous mins/week exceeding 960 min were excluded from analyses, (3) cases where participants indicated >7 days per week of activity were excluded from analyses, and (4) any single activity on a given day could not exceed 180 min. Decisions to exclude cases were made using Version 2.0 of the Guidelines for Data Processing and Analysis of the International Physical Activity Questionnaire (IPAQ)—Short Form (April 2004) (42). Activity levels <10 min per day were winsorized and set to 0.

We conducted analyses using R, version 4.2.0 (43). Our first aim was to evaluate differences in MVPA among demographic groups at baseline. We used Pearson correlations to measure associations between MVPA and age. For race/ethnicity groups, we collapsed the 7 response options into the following two categories: Black, Indigenous, and People of Color (BIPOC) and White. For gender identity groups, we collapsed the 11 response options into the following 3 categories: Man, Non-Binary, and Woman. We used an independent samples t-test to measure differences in MVPA between race/ethnicity groups. We used a Kruskal–Wallis test and *post hoc* test to determine which specific gender groups differed significantly in MVPA.

For our second aim, we used a Spearman rank correlation to measure associations between MVPA and depression symptoms and independent sample t-tests to test for differences between depression screening status and MVPA participation at baseline. Non-parametric tests were used when samples demonstrated significant skew.

For our third aim, we first used paired t-tests to evaluate changes in MVPA, depression symptoms, and the self-reported impact of mental health on physical activity stratified by baseline physical activity status (full sample, active at baseline, underactive at baseline). We also calculated the proportion of individuals who reported clinically significant improvement in depression in each of the baseline physical activity groups (full sample, active at baseline, underactive at baseline). Then we used both paired samples *t*-tests and proportions to examine changes in depression, as well as improvement and/or recovery in depression and maintenance of low depression among participants with different baseline levels of depression (positive or negative screening at baseline). Finally, we used both paired samples *t*-tests and proportions to examine changes in physical activity and improvement or maintenance in physical activity among participants with different baseline levels of physical activity (active at baseline, underactive at baseline). We considered hypothesis tests statistically significant using an alpha level of 0.05.

## Results

### Study participants

Of the 8786 individuals who were eligible and invited to participate, 950 agreed to participate and provided informed consent. Of this sample, 171 adults were excluded due to missing IPAQ data, and 24 adults were excluded due to outlier data, leaving a final sample of 755 adults (79.5%) at baseline. Participants were employed at 184 companies across a range of industries including technology (60.8%), care-related sectors such as healthcare and education (11.7%), labor-intensive fields such as manufacturing and transportation (8.9%), service industries such as hospitality and food service (7.9%), and other sectors not otherwise classified (9.8%). Seven hundred and three adults completed the follow-up survey. Of this sample, 124 adults were excluded due to missing IPAQ data and 24 adults were excluded due to outlier data, leaving a final sample of 555 (78.9%) at follow-up. Four hundred and eighty adults (63.6%) provided complete IPAQ data for both surveys. Baseline analyses were conducted using the sample of 755 adults, while longitudinal analyses were conducted using the sample of 480 adults.

We conducted an analysis of baseline differences between individuals who had complete IPAQ data in the follow-up survey (“completers”) and those who did not (“non-completers”) and found significant differences across 2 variables: PHQ-9 score and race/ethnicity (*p*s < 0.05). Baseline PHQ-9 scores were significantly lower among completers, and we observed a higher proportion of White-identifying participants among completers compared to non-completers. There were no significant differences in MVPA, age, or gender between completers and non-completers.

### Aim 1: baseline prevalence of depression symptoms and levels of physical activity

Of the 755 with valid baseline data, 47.3% (*n* = 357) of participants were deemed “underactive” according to U.S. guidelines (i.e., engaging in less than 150 min of MVPA per week). See Table 1 for a summary of the demographic characteristics and baseline descriptive statistics of the full sample, as well as the active and underactive subgroups.

**Table 1:** Demographic characteristics and descriptive statistics of sample at baseline in the full sample and stratified by baseline physical activity status.

	Full sample	Physically active	Physically underactive
	*N* = 755	*N* = 398	*N* = 357
Age			
Age (years), *M* *±* *SD*	33.6 ± 8.7	33.6 ± 8.8	33.6 ± 8.5
Gender Identity			
Woman, *N (%)*	430 (57.1%)	213 (53.5%)	217 (61.0%)
Man, *N (%)*	282 (37.5%)	163 (41.0%)	119 (33.3%)
Non-binary, *N (%)*	41 (5.4%)	20 (5.0%)	21 (5.9%)
Race/Ethnicity			
BIPOC, *N (%)*	329 (40.7%)	146 (36.7%)	160 (44.8%)
White, *N (%)*	446 (59.3%)	250 (62.8%)	196 (54.9%)
MVPA			
Minutes/week, *M* *±* *SD*	213.0 ± 233.5	339.7 ± 243.1	51.5 ± 46.8***
Depression Symptoms			
Total PHQ-9 score, *M* *±* *SD*	8.2 ± 5.4	7.3 ± 5.0	9.4 ± 5.7***
Positive screening, *N (%)*	285 (37.8%)	120 (30.2%)	165 (46.2%)***
Depression Severity Levels			
None-minimal (PHQ 0-4), *N (%)*	198 (26.2%)	119 (29.9%)	79 (22.1%)
Mild (PHQ 5–9), *N (%)*	253 (33.5%)	147 (36.9%)	106 (29.7%)
Moderate (PHQ 10–14), *N (%)*	159 (21.1%)	72 (18.1%)	87 (24.4%)
Moderately severe (PHQ 15–19), *N (%)*	79 (10.5%)	33 (8.3%)	46 (12.9%)
Severe (PHQ 20–27), *N (%)*	47 (6.2%)	15 (3.8%)	32 (9.0%)*

BIPOC, Black, Indigenous, and People of Color; MVPA, moderate-to-vigorous physical activity; PHQ-9, Patient Health Questionnaire-9.

* and *** indicate significance at the *p* = 0.05 and *p* = 0.001 level, respectively, between physically active and underactive samples.

There was a statistically significant difference in MVPA between men and women at baseline [*H*(2) = 9.1, *p* = 0.01] and *post-hoc* comparisons using Nemenyi's test of multiple comparisons revealed that men engaged in an average of 49 more minutes of MVPA per week than women. We also observed a significant gender difference in depression symptoms [*H*(2) = 17.0, *p* < .001]. *post-hoc* comparisons revealed a statistically—but not clinically—significant difference, with both women and adults identifying as gender non-binary reporting higher PHQ-9 scores than men (1.62 points and 2.68 points higher, respectively). Finally, there was a significant, negative correlation between age and depression symptoms (*r* = −.16. *p* < .001). There were no significant differences in PHQ-9 score across races and ethnicities (*p* = 0.85). There were no significant differences in MVPA based on other gender identities or races and ethnicities (*p*s > 0.07), and no significant correlation between MVPA and age (*p* = 0.43). While physical activity engagement and depression symptoms differed significantly between the active and underactive groups (see Table 1), the standard deviation within each group indicated substantial heterogeneity.

### Aim 2: baseline associations between physical activity and depression symptoms

Physical activity was negatively correlated with baseline PHQ-9 scores (*r* = −.19, *p* < .001). Individuals with a positive screening for depression engaged in less MVPA [*M* = 167.9 ± 201.3 min/week, *t*(734) = 3.6, *p* < .001] compared to those with a negative screening (*M* = 230.1 ± 240.1 min/week), though the standard deviations indicate substantial heterogeneity in the sample. The underactive group had significantly higher PHQ-9 scores [*M* = 9.8 ± 6.2, *t*(704.3) = 4.72, *p* < 0.001] than the active group (*M* = 7.7 ± 5.6).

### Aim 3: longitudinal changes in physical activity and depression symptoms

Participants were able to engage in any of the care modalities available on the platform (e.g., one-on-one teletherapy or telecoaching, self-guided digital resources). On average, participants engaged in 1.3 teletherapy sessions (range: 0-10), 1.4 telecoaching sessions (range: 0-12), and 2.6 self-guided digital resources (range: 0-19). Of the sample with complete data at 3 months, 76.5% (*n* = 367) were engaged with the platform at follow-up and 23.5% (*n* = 113) were not. Among the people engaged at 3 months, 77.4% (*n* = 284) engaged in at least one one-on-one provider session and 69.8% (*n* = 256) engaged with at least one self-guided digital resource. Additionally, 59% (*n* = 217) engaged in one modality of care (e.g., telecoaching only), 31.3% (*n* = 115) engaged in two modalities of care (e.g., teletherapy and digital resources), and 9.5% (*n* = 35) engaged in all three modalities of care.

See Table 2 for overall changes in MVPA, depression symptoms, and self-reported impact of mental health status on physical activity in the full sample and stratified by physical activity levels at baseline (active and underactive groups. Results show that MVPA significantly increased among the underactive subsample (by an average of 60 min/week). Depression scores decreased across the full sample and both active and underactive subsamples (though to a greater degree for the underactive subsample—reduction of 2.8 points). These levels of improvement in depression were statistically significant but potentially not clinically significant given the average change was less than the 5 points deemed clinically meaningful change on the PHQ-9. As a *post-hoc* analysis, the proportion of participants in each group who reported a decrease of at least 5 points on the PHQ-9 was analyzed: 27.7% of the full sample, 23.8% of the active group, and 32.7% of the underactive group experienced a clinically significant improvement in depression scores. The higher proportion of clinically significant improvement in the underactive group, as compared to the active group, highlights the interplay between depression and physical activity.

**Table 2:** Paired *t*-test results: changes in physical activity engagement, depression symptoms, and impact of mental health on physical activity stratified by baseline physical activity status.

	Baseline	Follow-Up	Difference	Confidence intervals	*t* (df)	*p*
	*M* (SD)	*M* (SD)	*M*	95% CI		
MVPA (mins/week)						
Full Sample	213.0 (233.5)	224.0 (243.2)	10.9	(−29.4, 7.5)	−1.2 (479)	0.25
Active	339.7 (243.1)	311.9 (269.4)	−27.8	(−0.8, 56.4)	1.9 (268)	0.06
Underactive	51.5 (46.8)	111.9 (140.6)	60.3	(−79.5, −41.2)	−6.2 (210)	<0.001***
PHQ-9 Score						
Full Sample	8.2 (5.4)	6.1 (4.5)	−2.2	(1.8, 2.6)	10.7 (479)	<0.001***
Active	7.3 (5.0)	5.6 (4.1)	−1.7	(1.2, 2.2)	6.8 (268)	<0.001***
Underactive	9.4 (5.7)	6.7 (4.9)	−2.8	(2.1, 3.4)	8.4 (210)	<0.001***
Impact of Mental Health on Physical Activity						
Full Sample	2.4 (1.3)	2.2 (1.2)	−0.2	(0.1, 0.3)	3.2 (478)	0.002*
Active	2.1 (1.1)	2.0 (1.1)	−0.1	(−0.04, 0.3)	1.4 (268)	0.15
Underactive	2.8 (1.3)	2.5 (1.3)	−0.3	(0.1, 0.5)	3.1 (209)	0.002*

*Ns* = 211–480. The active group was defined as MVPA ≥ 150 min/week. The underactive group was defined as MVPA < 150 min/week. MVPA, moderate-to-vigorous physical activity; M, mean; SD, standard deviation; 95% CI, 95% confidence intervals; PHQ-9, Patient Health Questionnaire-9. Impact of Mental Health on Physical Activity was measured with a single item assessing whether mental health had a negative impact on physical activity with higher scores indicating a greater negative impact.

* and *** indicate significance at the *p* = 0.05 and *p* = 0.001 level, respectively.

The self-reported impact of mental health on physical activity was the highest for the underactive group compared to the full sample or active group, indicating they perceived the most negative impact of their mental health challenges on their physical activity participation. The underactive group also saw the greatest improvement on this measure (average of 0.3-point reduction) which was statistically significant.

Table 3 details change in depression among participants with different levels of baseline depression. Participants with a positive depression screening at baseline (*n* = 178) reported an average decrease of 4.88 points on the PHQ-9, representing a statistically significant 35% reduction in depressive symptoms (*p* < .001). In addition, 66.9% of the sample reported improvement or recovery in depressive symptoms. Participants with a negative depression screening at baseline (*n* = 302) reported an average decrease of 0.57 points on the PHQ-9, representing a statistically significant 12% reduction in depressive symptoms (*p* = 0.004). Among this sub-sample, 94% maintained their negative screening for depression at follow-up.

**Table 3:** Changes in depression, improvement and/or recovery in depression, and maintenance of low depression among participants with different levels of baseline depression.

	*n*	Improvement	Recovery	Improvement and Recovery	Improvement or Recovery	Maintenance	Baseline	Follow-Up	Difference	Cohen’s d	df	*t*	*p*
		*n* (%)	*n* (%)	*n* (%)	*n* (%)	*n* (%)	*M* (SD)	*M* (SD)	*M*				
Depression Symptoms—Positive Screening (PHQ-9 ≥ 10)	178	96 (53.9%)	99 (55.6%)	76 (45.7%)	119 (66.9%)	–	14.01 (3.53)	9.13 (4.61)	4.88	1.18	177	13.83	<.001***
Depression Symptoms—Negative Screening (PHQ-9 < 10)	302	–	–	–	–	284 (94.0%)	4.84 (2.86)	4.27 (3.33)	0.57	0.18	301	2.90	0.004***

*Total N* *=* *480;* Depression was measured with the PHQ-9. Improvement was defined as a participant's score decreasing by 5+ points at follow-up. Recovery was defined as having a positive screening for depressive symptoms at baseline and a negative screening at follow-up. Maintenance was defined as having a negative screening for depressive symptoms at both baseline and follow-up. M, mean; SD, standard deviation; df, degrees of freedom.

*** indicates significance at the *p* = 0.001 level.

Table 4 reports changes in physical activity among participants with different levels of baseline physical activity. Participants who were classified as underactive at baseline (*n* = 211) reported an average increase of 60.33 min/week in activity, representing a statistically significant 117% increase in MVPA (*p* < .001). In addition, 27.5% of this sample improved their level of physical activity enough to be classified as active at follow-up, though there was considerable heterogeneity in this group. Participants who were classified as active at baseline (*n* = 269) reported no statistically significant change in their activity at follow-up and 78.4% maintained their classification as active.

**Table 4:** Changes in physical activity and improvement or maintenance in physical activity among participants with different levels of baseline physical activity.

	*n*	Improvement	Maintenance	Baseline	Follow-Up	Difference	Cohen’s d	df	*t*	*p*
		*n* (%)	*n* (%)	*M* (SD)	*M* (SD)	*M*				
MVPA—underactive Group (<150 min/week)	211	58 (27.5%)	–	51.54 (46.75)	111.87 (140.64)	60.3	−0.58	210	−6.21	<.001***
MVPA—active Group (≥150 min/week)	269	–	211 (78.4%)	339.71 (243.10)	311.91 (269.36)	−27.8	0.11	268	1.91	0.06

*Total N* *=* *480;* Physical activity was measured with the IPAQ. Improvement in MVPA was defined as having an MVPA score classified as underactive at baseline and active at follow-up. Maintenance in MVPA was defined as having an MVPA score classified as active at both baseline and follow-up. MVPA, moderate-to-vigorous physical activity; M, mean; SD, standard deviation; df, degrees of freedom.

*** indicates significance at the *p* = 0.001 level.

## Discussion

We examined whether MVPA and depression symptoms changed over a three-month period among adults with access to a multi-modal digital mental health platform and observed positive changes across multiple domains of health. At baseline, adults with higher depression scores were more likely to be underactive, as defined by the U.S. Department of Health and Human Services physical activity guidelines. Participants with elevated depression reported significant improvements in depression symptoms, while nearly all with low baseline symptoms remained asymptomatic. On average, individuals classified as underactive at baseline increased their physical activity over time, whereas those who were active at baseline maintained high levels of activity throughout the study period.

Our first aim was to examine the baseline prevalence of depression symptoms and physical activity engagement in the sample. There were no significant differences in baseline physical activity participation across ages or races/ethnicities. However, men reported significantly higher physical activity than women, consistent with prior research showing lower activity levels among adult women (44). This gender gap may be explained by barriers such as sociocultural norms, role expectations, and lower self-efficacy and social support for physical activity (45–47).

We observed a negative relationship between age and depression symptoms in our study which is echoed in prior literature showing that younger adults report more depression symptoms than middle-aged adults (48). This pattern may reflect challenges more common in young adulthood, such as life transitions, identity formation, and financial struggles (48, 49). Participants identifying as women or gender non-binary reported significantly higher PHQ-9 scores than those identifying as men. Prior research also shows higher rates of depression symptoms in women and gender minorities, with the latter group reporting up to three times greater odds of depression and anxiety compared to their cisgender counterparts (50, 51). These disparities may be driven by systemic discrimination, prejudice, and minority stressors often experienced by gender minorities (52, 53).

Our second aim was to examine baseline associations between physical activity and depression symptoms. MVPA was negatively correlated with depression scores, such that individuals with higher depression symptoms reported lower levels of physical activity. This association may be explained by certain depression symptoms relevant to physical activity participation, such as anhedonia, sleep disturbance, fatigue, and low motivation (54, 55). Participants screening positive for depression on the PHQ-9 reported approximately 62 fewer minutes of MVPA per week than those screening negative. Active participants reported significantly lower depression scores than underactive participants, supporting prior research on the bidirectional link between physical activity and depression symptoms (7).

Our third aim was to examine changes in depression symptoms and MVPA over the 3-month follow-up period. Among participants with a positive depression screening at baseline, 66.9% improved or recovered in their depressive symptoms, with an average PHQ-9 reduction of almost 5 points, meeting the threshold for clinically meaningful improvement. We also examined whether people with a negative depression screening at baseline maintained their mental health status over time. This group reported a small, but significant, decrease in their PHQ-9 scores (mean reduction = 0.57 points) at follow-up and 94% maintained their low-risk status. These findings align with and add to a myriad of prior research demonstrating that digital mental health platforms can support both symptom improvement and maintenance of positive mental health outcomes (32, 56, 57).

Participants who were underactive at baseline reported a 117% increase in weekly physical activity at follow-up, from 52–112 min–an average gain of 60 min per week. Over one-quarter (27.5%) of this group shifted from underactive to active classification at follow-up. Although these improvements are promising, the group's average activity at follow-up remained below the recommended 150 min of MVPA per week, likely because the platform was not explicitly designed to promote physical activity. Nonetheless, research shows that underactive individuals can achieve meaningful health benefits by increasing activity from baseline levels, even without reaching guideline thresholds (58).

Among participants classified as active at baseline, 78.4% maintained their active status at follow-up, with no significant change in weekly MVPA. Although we observed a slight, non-significant decrease of 28 min per week, the group's average MVPA remained high at 311 min per week. This group likely included individuals with established exercise routines who sustained their habits over the study period. The platform's digital resource library included psychoeducational content on habit formation and routine-building, which may have supported continued activity. Given links between habit formation and physical activity (59), this content may have contributed to maintaining or enhancing physical activity engagement. Additionally, over 40% of participants engaged in two or more care modalities, suggesting that tailored, multi-modal support may have reinforced physical activity behaviors. Employer-sponsored platforms offering integrated mental and physical health resources may create a more supportive environment for sustaining health behaviors.

These findings contribute to growing evidence that digital, remotely delivered interventions are scalable and may be particularly beneficial for underactive adults seeking mental health support or aiming to increase physical activity (31). Notably, underactive participants, but not active ones, reported a significant reduction in the extent to which mental health symptoms interfered with physical activity over time. This change may reflect improvements in psychological factors relevant to physical activity, potentially driven by engagement in teletherapy, telecoaching, psychoeducation, and/or self-guided care.

### Limitations

This study was observational in nature, meaning that while we were able to observe that depression symptoms and MVPA improved over time, we were unable to determine the temporal sequence of the changes. A prospective, experimental design and random assignment to treatment conditions is needed to test the directionality and causality of these improvements. We also designed a single-item, self-report question to assess the impact of mental health issues on physical activity participation. Though it was based on an item from the 36-item Medical Outcomes Study (MOS) Short Form Survey, this item has yet to be validated and may be inaccurately measuring the barrier that mental health may pose to participation in physical activity. Further, we used the widely accepted PHQ-9 cut-off of 10 to define positive depression screening, and future research could examine whether results differ when alternative cut-off thresholds or severity categories are applied. Next, although participants were drawn from a range of industries, we did not examine whether outcomes varied by industry sector; future research could explore how occupational context may influence engagement with digital mental health tools and subsequent behavior change. We also did not assess implementation-related factors (e.g., feasibility, organizational engagement, communication strategies), which are critical considerations for scaling digital mental health interventions and should be a focus of future research. Finally, we observed a higher proportion of White-identifying participants among completers of the follow-up survey, compared to non-completers. This, paired with the fact that platform access was gated by employer-sponsorship, limits the generalizability of our findings.

## Conclusions

These findings highlight the beneficial impact of a novel, multi-modal digital mental health platform in bringing about positive change in distinct, but related, domains of health. Digital mental health programs that focus on single domains of health and provide limited treatment modality options are limiting both their reach and impact. By personalizing care and providing individuals with several choices of treatment modalities across multiple domains of health, we observed a statistically significant reduction in depression symptoms, as well as an overall increase in weekly physical activity engagement over a three-month period. Individuals screening positive for depression at baseline demonstrated a reduction of almost 5 points on the PHQ-9, approaching clinical significance, while individuals not achieving sufficient levels of physical activity at baseline more than doubled their weekly engagement levels during the follow-up period. Our findings underscore the need for digital health platforms to broaden their focus beyond single domains and adopt comprehensive, multi-modal treatment models that can effectively address the complex, and often, interconnected health challenges of individuals seeking care. Given the observational nature of the present study, future investigations should incorporate an experimental design to determine the causality of improvement in physical activity engagement and depressive symptoms. Moreover, future investigations should utilize a longer-term follow-up period (perhaps 6 or 12 months) to assess whether the changes demonstrated in the present study can be maintained over time, given the relatively short duration of the present study.

## Data Availability

The datasets presented in this article are not readily available. Individual de-identified data may be shared privately for research purposes upon receipt of a methodologically sound proposal, and whose proposed use of the data from the study related to this article are approved by the authors. To gain access, requesters will need to sign a data access agreement that includes a commitment: (1) to use the data only for research purposes; (2) to not attempt to, or actually, re-identify any individual; (3) to secure the data using appropriate safeguards; and (4) to destroy or return the data after analyses are completed. Requests may be sent to the corresponding author. Requests to access the datasets should be directed to Sara Sagui Henson, research@joinmodernhealth.com.
