# Correction: Improvements in physical activity and depression symptoms: an observational study of users of a multi-modal digital mental health platform

**DOI:** 10.3389/fdgth.2025.1755165

**Published:** 2025-12-09

**Authors:** Camille E. Welcome Chamberlain, Shannon Lindsay, Brooke J. Smith, Sara Sagui Henson, Cynthia Castro Sweet, Sara M. Levens

**Affiliations:** 1 Clinical Strategy and Research Team, Modern Health, San Francisco, CA, United States; 2 Department of Psychological Science, University of North Carolina at Charlotte, Charlotte, NC, United States

**Keywords:** digital health, mental health symptoms, physical activity, depression, technology

There was a mistake in a percentage reported in the abstract. The sentence was displayed as “Physical activity participation improved by 217% among individuals deemed underactive at baseline (*p* < 0.001), while individuals who were active at baseline maintained high levels of physical activity (*p* = 0.06).”

A correction has been made to the abstract, in the last sentence of the results:

“Approximately 47% of participants were physically underactive at baseline, defined as <150 min of physical activity per week. Men participated in more physical activity than women (*p* = 0.005), while women and individuals identifying as gender non-binary reported more depression symptoms than men (*p*s < 0.05). Older adults reported fewer depression symptoms than younger adults (*r* = −0.16, *p* < 0.001). Baseline MVPA baseline was negatively correlated with depression symptoms (*r* = −0.19, *p* < 0.001). Depression scores significantly improved, with 66.9% of adults at risk of depression improving or recovering (*p* < 0.001) and 94% of adults with low depression symptoms maintaining this status over time (*p* = 0.004). Physical activity participation improved by 117% among individuals deemed underactive at baseline (*p* < 0.001), while individuals who were active at baseline maintained high levels of physical activity (*p* = 0.06).”

The original version of this article has been updated.

